# The Effect of Oral Simvastatin on the Clinical Outcome of Patients with Severe Traumatic Brain Injury: A Randomized Clinical Trial

**DOI:** 10.4314/ejhs.v31i4.15

**Published:** 2021-07

**Authors:** Sajad Shafiee, Alireza Zali, Misagh Shafizad, Amir Emami Zeydi, Saeid Ehteshami, Fatemeh Rezaii, Abbas Tafakhori, Abolhasan Ertiaei, Hadi Darvishi-Khezri, Mohammad Khademloo, Reza Jalili Khoshnood

**Affiliations:** 1 Department of Neurosurgery, Orthopedics Research Center, Mazandaran University of Medical Sciences, Sari, Iran; 2 Functional Neurosurgery Research Center, Shohadaye Tajrish Neurosurgical Center of Excellence, Shahid Beheshti University of Medical Sciences, Tehran, Iran; 3 Department of Medical-Surgical Nursing, Nasibeh School of Nursing and Midwifery, Mazandaran University of Medical Sciences, Sari, Iran; 4 Student Research Committee, Mazandaran University of Medical Sciences, Sari, Iran; 5 Iranian Center of Neurological Research (ICNR), Neuroscience Institute, Tehran University of Medical Sciences, Tehran, Iran; 6 Department of Neurosurgery, Imam Khomeini Hospital, Tehran University of Medical Science, Tehran, Iran; 7 Thalassemia Research Center (TRC), Hemoglobinopathy Institute, Mazandaran University of Medical Sciences, Sari, Iran; 8 Department of Community Medicine, Faculty of Medicine, Mazandaran University of Medical Sciences, Sari, Iran

**Keywords:** Simvastatin, Brain Injuries, Traumatic, Glasgow Outcome Scale, Patient Outcome Assessment

## Abstract

**Background:**

Despite recent promising pharmacological and technological advances in neurosurgical intensive care, the overall TBI-related mortality and morbidity remain high and still pose a major clinical problem. The aim of this study was to evaluate the effect of oral simvastatin on the clinical outcome of patients with severe TBI.

**Methods:**

In a double-blind placebo-controlled randomized clinical trial a total of 98 patients with severe TBI in Imam Khomeini Hospital in Sari, Iran, were evaluated. Patients who meet the inclusion criteria were randomly allocated into two groups (n=49). In addition to supportive therapies, the intervention group received oral simvastatin (40 mg, daily) for 10 days, and the control group received the placebo (10 days). Patients' Glasgow coma scale (GCS) score, in hospital mortality, duration of mechanical ventilation and length of ICU and neurosurgery ward stay were evaluated during three-time intervals (T1: admission, T2: discharge and T3: one month after discharge).

**Results:**

The percentage of conscious patients was 18.9% (7 cases) in the simvastatin group and 3.1% (1 case) in controls (P=0.06) at T2. One month after discharge (T3) the proportion of conscious patients significantly increased in the simvastatin group compared to control group (64.9 % versus 28.1 %; P=0.002). There was no significant difference for the mean of GCS score between the simvastatin group and control group at T1 (6.41 ± 1.30 versus 6.41 ± 1.28, respectively; P = 0.98). However, the mean score of GCS in patients who received simvastatin was significantly greater than controls at T2 and T3 (p<0.05). There was no significant differences between two group in-terms of length of mechanical ventilation, ICU and neurosurgery ward stay.

**Conclusion:**

According to the results of this study it seems that using simvastatin may be an effective and promising therapeutic modality for improving GCS score during TBI recovery.

## Introduction

Traumatic Brain Injury (TBI) is the leading cause of neurological morbidity and mortality worldwide (1–3). Many of TBI survivors will experience short and long-term TBI-related disabilities and complications, which imposes huge clinical, social and economic burdens on the healthcare system and society (4–6). Despite recent promising pharmacological and technological advances in neurosurgical intensive care, the overall TBI-related mortality and morbidity remain high and still pose a major clinical problem. To date, no pharmacological intervention, with strong evidence, is available to clearly improve the outcome of patients with severe TBI (2,7). Therefore, trying to evaluate the overall clinical efficacy of new therapeutic modalities on the clinical outcomes of patients with severe TBI is necessary (8).

Although the exact pathophysiological mechanism of TBI is not fully elucidated, neuroinflammation has been proposed as a highly plausible mechanism. Typically, after TBI a large amount of cytokines and chemokines will be released and an acute inflammatory response occurs in the central nervous system that can exacerbate the damage caused by TBI (4, 9). Theoretically, limiting neuroinflammation after head trauma can lead to reduced mortality and disability, which support the promising potential benefit of anti-inflammatory agents in treatment of patients with TBI (10–11). There is a growing body of evidence that confirms the anti-inflammatory properties of statins, besides their cholesterol-lowering effect (5, 12–14). Statins can increase neurogenesis, suppress apoptosis, reduce microglial activity and ultimately reduce inflammation-induced astroglial activation (13, 15–16). In addition, it was shown that statins have beneficial effects on neurological diseases such as Alzheimer's disease (17–18) and Parkinson's disease by modulating inflammation (19–21). Several animal studies, demonstrated a neuroprotective and also a significant positive effects of statins on TBI-induced inflammation (22–23). However, very few human studies have been conducted to evaluate the efficacy of statins in patients who suffered from TBI, with conflicting results. One study suggested the potential benefit of statins in patients with TBI, while this finding was not confirmed by other studies (24–26).

Due the paucity of information and few small studies with conflicting results regarding the efficacy of statins in patients with TBI, the aim of this study was to evaluate the effect of simvastatin on the clinical outcomes of patients with severe TBI.

## Methods

**Study design and sample**: In a double-blind, randomized clinical trial, a total of 98 patients with severe TBI who were hospitalized in Imam Khomeini Hospital, Sari, Iran, were enrolled between October 2018 and February 2019.

Patients who meet the inclusion criteria were randomly allocated to two equally sized groups (intervention and control). In addition to supportive therapies, patients in intervention group received the oral simvastatin (Poursina Pharmaceutical Co. Tehran-Iran; 40 mg, daily) for 10 days, and the control group received the placebo (10 days) in addition to supportive therapies. Simvastatin or placebo was administered through nasogastric tube. The safety profile of simvastatin at doses up to 40 mg has been well-documented (27)

**Inclusion and exclusion criteria**: The inclusion criteria were aged 18–60 years, no allergy to statins, non-use of NSAIDs, corticosteroids, statins, severe brain injury with glasgow coma scale (GCS)≤8 when presenting to the emergency department, no intracranial lesions in the brain CT scan requiring neurosurgical intervention, no history of autoimmune, cardiac, respiratory, neuromuscular, hepatic, or renal diseases. Patients with GCS score >8, simultaneous injury to other organs that required surgical intervention, presence of sepsis during the first 72 hours of admission to hospital, and history of drug poisoning were excluded from the study.

**Randomization and blinding**: Patients who fulfilled the inclusion criteria, were randomly allocated into two equally sized groups, using a computer-generated list of random numbers by a nurse who was unaware of the study groups. Also, therapists were unaware of how the patients were divided into the groups. Patients' outcomes were evaluated by an ICU nurse who was blinded to the study group.

**Data collection:** Data were collected using a researcher-made checklist that includes patients' demographic and clinical characteristics such as age, sex, occupation, chronic illness mechanism of trauma and GCS score that was evaluated at the admission, discharge and one month later. Also, duration of mechanical ventilation, and length of ICU and neurosurgery ward stay were measured.

**Outcomes**: The primary outcome of this study was changes in patients' GCS score during the study period at three times: at admission [T1], discharge [T2] and one month after discharge [T3]. The main secondary outcomes were, in hospital mortality, duration of mechanical ventilation and length of ICU and neurosurgery ward stay.

**Ethical consideration**: This study was conducted after obtaining the approval of institutional ethics committee. In this study, the researchers received the informed consent from a surrogate decision maker of all participants, after explaining the aim of the study. The study was registered in the Iranian Registry of Clinical Trials Database (IRCT20180802040668N1).

**Sample size**: We performed power calculation for our study. The power (1-β) was estimated around 0.98 by G*Power software 3.0.10 for current study with α=0.05, total sample size=98, effect size=0.4 and the correlation among repeated measures=0.8 when the GCS score was the dependent variable.

**Statistical analysis**: Descriptive statistics were expressed as means ± standard deviation and/or median (interquartile range) and/or frequency (percentage) where appropriate. All quantitative data were tested for normality using the Shapiro-Wilk test. The two groups were compared in terms of baseline characteristics using Student's t-test for age and the chi-square or Fisher's exact test for dichotomous variables.

The comparison of the mean score of GCS was fulfilled with Mann-Whitney U-test between the simvastatin and control groups at each time points (T1 to T3). Additionally, we categorized the score of GCS as mild, moderate and severe to calculate a risk ratio (RR) to estimate the efficacy of the treatment. A severe TBI has been defined as GCS 3–8, a moderate injury as GCS 9–12, a mild injury as GCS 13–14, a conscious GCS 15. We used a generalized estimating equation (GEE) model to examine changes in GCS score after adjusting for sex among the simvastatin and control groups from baseline to the end of study. The incidence of mortality and its 95% confidence interval (CI) was calculated using the binomial exact method in STATA software. A Mann-Whitney U-test was applied alongside an estimation of Cohen's d to compare secondary outcomes between the groups of the study.

All statistical tests were two-tailed, and a P<0.05 was considered statistically significant. Data were analyzed using the SPSS software package (version 16.0, SPSS Inc., Chicago, IL, USA) and STATA version 13.0 (Stata Corp, College Station, TX, USA). GEE and making a plot were also carried out by Minitab software 13.0 (Minitab Inc, State College, PA, USA).

## Results

**Figure 1:**
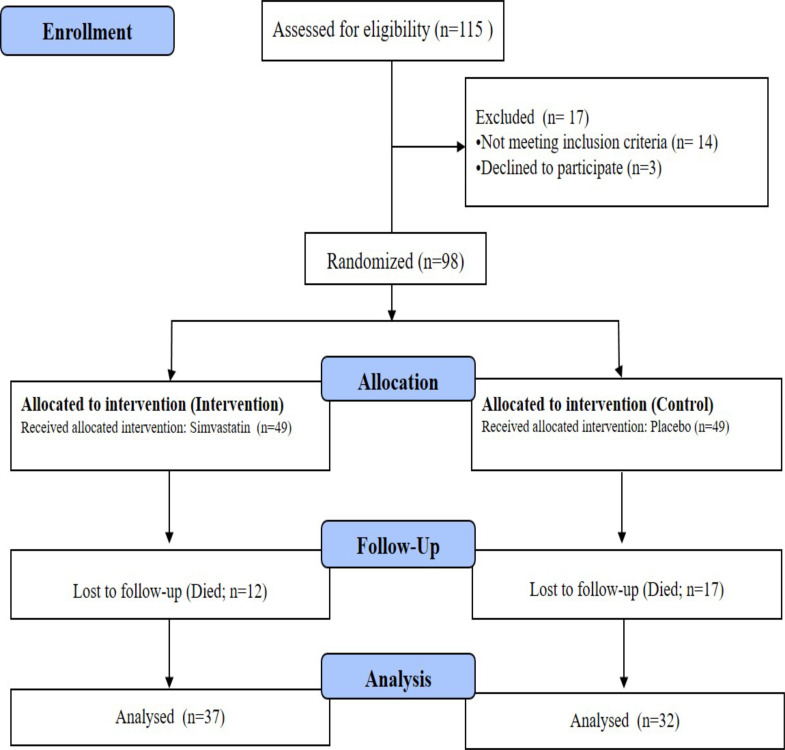
Flow chart of the study

Ninety eight patients were enrolled in the study at T1 (admission time). Thirty seven patients completed the study at T2 (discharge) and T3 (one month after discharge) in simvastatin group. The number of patients who ended the study in the control group was 32 at time points T2 and T3 (Figure 1).

Basic demographic and clinical characteristics of the included patients have been shown in Table 1.

**Table 1:** Basic demographic and clinical characteristics of the patients in both groups

		Group		p-value
			
		Simvastatin (n = 49)	Control (n = 49)	
Age, year		38.0 ± 13.0	36.6 ± 11.4	0.57^a^
Sex	Male	32 (65.3)	34 (69.4)	0.83^b^
	Female	17 (34.7)	15 (30.6)	
Trauma mechanism	Auto accident	18 (36.7)	17 (34.7)	0.91^b^
	Motorcycle accident	15 (30.6)	14 (28.6)	
	Pedestrian	16 (32.7)	18 (36.7)	
Vegetative state		8 (16.3)	9 (18.4)	1^b^

Data are presented as mean ± standard deviation or number (percentage).

a p-value was obtained with Student's t-test.

b p-value was obtained with chi-square test.

**Primary outcome**

**Glasgow coma scale (GCS) and severity of traumatic TBI**: All admitted patients to the ICU had severe TBI. The percentage of conscious patients was 18.9% (7 cases) in the simvastatin group and 3.1% (1 case) in controls (P=0.06) at T2. At the end of study (T3) the proportion of conscious patients significantly increased in the simvastatin group compared to control group (64.9 % [24 cases] versus 28.1 % [9 cases]; P=0.002).

At T2 (discharge), the percentage of patients with mild TBI was 24.32% (9 cases) in simvastatin group compared to 6.25% (2 cases) in control group (P = 0.02). At T3 (one month after discharge), the percentage of patients with mild TBI decreased to 10.81% (4 case) in the patients under simvastatin therapy. However, the proportion of mild TBI patients had a raise in control group at T3 (37.5%, 12 cases). At T3, the difference of patients with mild TBI between two groups was not significant (P=0.21). At time point T2, the percentage of patients with mild to moderate TBI in simvastatin and control groups were 81.08% (30 cases) versus 96.88% (31 cases), respectively; P = 0.06. A reduction in patients with mild to moderate TBI was found after simvastatin treatment and control at T3 time point, 35.13% (13 cases) in simvastatin groups in comparison with 71.87% (23 cases) in control group (P=0.002).

At T2, the patients with moderate severity of TBI in simvastatin group was 56.76% (21 cases) compared to 90.62% (29 cases) in control group (P=0.002). At T3, 24.32 percent (9 cases) of patients under treatment with simvastatin linger to moderate TBI state. However, the proportion of moderate TBI state in control group was 34.37% (11 cases) (P=0.35). There were not patients with severe TBI at T2 and T3 in the simvastatin and control groups. The severity of TBI at all-time points (T1 to T3) in both groups are provided in Table 2.

**Table 2:** Severity of TBI at time points in both the groups

	T1		T2		T3	
	
	Simvastatin n = 49	Control n = 49	Simvastatin n = 37	Control n = 32	Simvastatin n = 37	Control n = 32
Conscious	0 (0)	0 (0)	7 (18.9)	1 (3.1)	24 (64.9)	9 (28.1)
Mild	0 (0)	0 (0)	9 (24.32)	2 (6.25)	4 (10.81)	12 (37.5)
Mild to moderate	0 (0)	0 (0)	30 (81.08)	31 (96.88)	13 (35.13)	23 (71.87)
Moderate	0 (0)	0 (0)	21 (56.76)	29 (90.62)	9 (24.32)	11 (34.37)
Severe	49 (100)	49 (100)	0 (0)	0 (0)	0 (0)	0 (0)

T1: At admission, T2: At discharge, T3: At one month after discharge, ^η^ There were not any patients with severe TBI.

**Figure 2:**
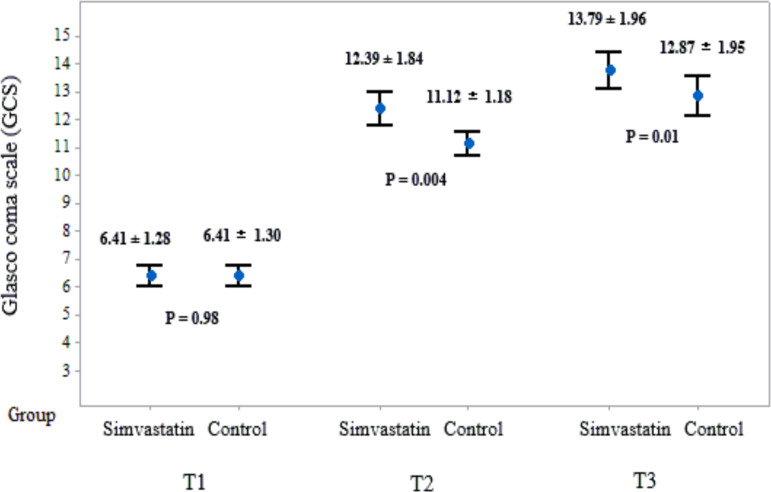
Changes of mean score of GCS in patients in two groups CI: confidence interval, GCS: Glasgow coma scale, T1: At admission, T2: At discharge, T3: At one month after discharge Data are presented as mean ± standard deviation. P-values were obtained with Mann-Whitney U test after

There was no significant difference for the mean of GCS score between the simvastatin group and control group at T1 (6.41 ± 1.30 versus 6.41 ± 1.28, respectively; P = 0.98). The mean score of GCS in patients under treatment with simvastatin significantly was greater than controls at T2 and T3 (Figure 2).

After controlling the sex's effect, GEE analyses revealed that, overall, GCS score were higher in the simvastatin group than in the control group (coefficient=- 0.45, standard error: - 0.23, P=0.04, adjusted R2: 0.81%).

## Secondary Outcomes

**Length of mechanical ventilation (MV)**: The median (interquartile range) of length of MV was 9 days (4 – 14) in the simvastatin group compared to 11 days (5 – 14) in the controls (P = 0.69). The estimated Cohen's d for the length of MV between the groups was - 0.08, 95% CI -0.47 to 0.32.

**Length of ICU stay**: The median (interquartile range) of length of ICU stay was 10 days (5 – 15) in the simvastatin group compared to 12 days (8 – 16) in the controls (P = 0.26). The estimated Cohen's d for the length of ICU stay between the groups was - 0.23, 95% CI - 0.62 to 0.17.

**Length of neurosurgery ward stay**: The median (interquartile range) of length of neurosurgery ward stay was 5 days (4 – 7) in the simvastatin group compared to 6.5 days (4.5 – 9.5) in the controls (P = 0.30). The estimated Cohen's d for the length of neurosurgery ward stay between the groups was - 0.24, 95% CI -0.64 to 0.15.

**Mortality**: The incidence of mortality was 24.49 per 100 cases (95% CI 12.01 to 36.97) in simvastatin group and 34.69 per 100 cases (95% CI 20.88 to 48.51) in control group; 11 cases in simvastatin group versus 17 cases in controls (P=0.26). Additionally, no significant adverse effects were observed and reported in this study.

## Discussion

The results of this study showed that simvastatin had a significant effect in improving GCS scores of patients with severe TBI. Also, patients who received simvastatin had a non-significant lower rate of mortality, length of mechanical ventilation, ICU and neurosurgery ward stay, compared to control group.

Previous animal studies demonstrated the anti-inflammatory, pleiotropic and neuroprotective effects of statins after severe TBI (28–30). Also, they were also less likely to lose tissue and brain function, which could ultimately improve post-TBI outcomes (31). On the other hand, the results of animal study in Taiwan demonstrated the positive neuroprotective effect of simvastatin with antioxidants combination (23). In contrast, the results of a study in patients with subarachnoid hemorrhage do not support a beneficial effect of simvastatin in these patients (32). The possible explanation for this discrepancy may be due to differences in the study population, statin type, and prescribed dose. In line with the results of this study, Naghibi et al. showed that patients who suffered from TBI and received simvastatin had a higher GCS score at discharge in comparison with control group (24). In the present study, patients who received simvastatin showed a significantly higher score of GCS during discharge and one month after discharge than the placebo group, which was consistent with a study in the United States (33). In an animal experiment, Abrahamson et al. showed that simvastatin therapy can effectively decrease post-injury beta-amyloid peptide levels and ameliorate pathological squeal of TBI (34). In another in-vivo study it has been shown that simvastatin significantly attenuates TBI-induced depression-like behavior via its antineuroinflammation properties in the hippocampus (35). The results of a study by Lu et al. indicated that using either simvastatin or atorvastatin in TBI rat model, can significantly promote neurogenesis and TBI-induced angiogenesis, enhance spatial learning, and decrease neuronal loss, with the superior therapeutic benefits of simvastatin (36). However, the results of an observational study did not support the efficacy of statin use in patients with moderate to severe TBI (16).

The results of our study showed that, although the simvastatin group had a 2 days shorter length of ICU stay compared to control group, this difference was not statistically significant. This finding is consistent with the results of other studies that demonstrated the beneficial effects of statin in reducing the length of critically ill ICU patients (24, 33, 37). The length of ICU stay greatly depends on a variety of factors, including neurological status, nosocomial infections, multi-organ failure, and previous respiratory failure that requires mechanical ventilation. Therefore, it is difficult to reliably determine whether statin use reduces or increases the length of stay in the ICU (33).

In our study patients who received simvastatin had a lower mortality rate in comparison with control group. However, the differences was not statistically significant. Contrary to the results of the present study, Lokhandwala et al. (33) and Khokhar et al. (38) showed that using statins has a significant relationship with reducing mortality in patients with TBI. Based on previous evidence, in justifying this difference, statins are expected to be associated with an increase in patients' GCS scores, improved mental status, improved neurological, mental and memory status, and consequently reduced patient mortality in the ICU. But some factors such as a history of previous underlying illnesses, the severity of the initial injury, the likelihood of involvement with nosocomial infections, and other complications of the disease process can in part explain the different prognosis (33). On the other hand, another important factor is the effect of previous use of statins due to previous underlying disease. The results of a study showed that older adults with TBI who were treated with statins previously had a 76% lower risk of in hospital mortality and 13% higher functional recovery (39). Therefore, it is recommended to consider the previous consumption of statins and other confounding factors in future studies. No significant side effects have been reported in our study. It has been previously indicated that statins are usually well tolerated by the patients, with an excellent safety profile and no significant side effects, especially in short term use (40–41). Lack of information about other measures for evaluating patients' neurologic function, such as modified Rankin Scale (mRS), Glasgow Outcome Scale (GOS), and Barthel Index (BI), and also pattern of intracranial injury are some limitations of this study that should be considered.

In conclusion, according to the results of this study it seems that using simvastatin is an effective, well tolerated, relatively safe and easy administration modality for improving GCS score during TBI recovery.
